# Scheduling of methylphenidate: Preventing misuse or impeding ADHD treatment adherence?

**DOI:** 10.4102/sajpsychiatry.v30i0.2335

**Published:** 2024-09-24

**Authors:** Renata Schoeman, Sophia Weinberg

**Affiliations:** 1 Stellenbosch Business School, Bellville, South Africa

**Keywords:** attention deficit hyperactivity disorder, treatment adherence, barriers to care, methylphenidate, diversion, stakeholder interviews, regulatory

## Abstract

**Background:**

Attention-deficit hyperactivity disorder (ADHD) is a common neurodevelopmental disorder, with a chronic, and potentially debilitating course if untreated. Medication adherence is poor – negatively affecting emotional, social, educational and employment outcomes. The current Schedule 6 status of methylphenidate (MPH) drives healthcare resource utilisation and costs – a potential barrier to care.

**Aim:**

This study explored stakeholders’ understanding and perceptions of the potential impact of a regulatory shift in the scheduling of MPH on treatment accessibility and adherence for ADHD.

**Setting:**

Participants from multiple stakeholder groups, involved in ADHD management in South Africa, were recruited via professional networks.

**Methods:**

A qualitative analysis of semi-structured interviews with 23 stakeholders was conducted to explore their views on the utility, benefits and risks associated with rescheduling MPH.

**Results:**

Six key themes emerged from the interviews: ‘adherence’, ‘accessibility’, ‘affordability’, ‘stigma’, ‘rescheduling of MPH’ and ‘risk mitigation’. Core to these themes is the role of the scheduling of MPH – which can have a protective societal role, but also acts as a barrier to care for individuals with ADHD.

**Conclusion:**

The current Schedule 6 status of MPH is not an effective strategy to prevent misuse and diversion but negatively impacts on treatment adherence. The positive outlook from stakeholders on rescheduling MPH holds significant implications for the ADHD landscape in South Africa.

**Contribution:**

It is crucial to address stigma, facilitate fundamental change in service delivery and remove structural and practical barriers to care to improve outcomes for individuals with ADHD. A framework for ADHD treatment adherence is provided.

## Introduction

Medication adherence is a complex issue that affects the successful management of chronic conditions such as attention-deficit hyperactivity disorder (ADHD). Non-adherence to ADHD medication is reported to be high – ranging from 13.2% to 64%.^1^ Yet, adherence is considered the single most modifiable factor associated with treatment outcomes.^2^

Attention-deficit hyperactivity disorder can have significant negative impacts on individuals’ emotional, social and economic wellbeing.^3^ This noted impact underscores the importance of early diagnosis and effective management of ADHD to reduce its economic burden on individuals, families and society. Consistent with international guidelines, South African guidelines advocate for an individualised approach to the management of ADHD, with psychostimulants – specifically methylphenidate (MPH) – remaining the gold standard of psychopharmacological intervention.^4^

High medication costs are one factor that can pose a significant barrier to adherence for individuals with ADHD, especially those with low incomes and inadequate insurance coverage.^5^ In South Africa, MPH is considered a Schedule 6 controlled substance – a strategy considered crucial in preventing drug abuse and diversion, ensuring the integrity of controlled substances.^6^ However, this level of control undoubtedly contributes to increased healthcare resource utilisation and costs as monthly scripts are required, and dispensing is limited to a 30-day supply of medication. However, there is no research on the potential or real impact of rescheduling of MPH on treatment outcomes and diversion of medication.

This study explored the perceived potential impact of a regulatory shift in the scheduling of MPH on treatment accessibility and adherence from the unique vantage points of diverse stakeholders involved in ADHD management. Understanding the impact of the regulatory environment as a barrier to care in terms of treatment accessibility and adherence could guide policymakers, healthcare providers (HCPs) and other stakeholders in identifying contextually relevant regulations for MPH to improve the outcomes of individuals with ADHD, while considering societal impact.

## Literature review

Attention-deficit hyperactivity disorder is a common neurodevelopmental disorder with a global prevalence estimate of 7.6% in children aged 3–12 years of age and 5.6% in adolescents.^7^ In adults, the estimated global prevalence rate of ADHD is 2.8%.^8^ Data regarding prevalence rates in South Africa are limited but have been estimated to be approximately 2.5% in children and 3% in adults.^9,10^

Attention-deficit hyperactivity disorder is a costly, chronic disorder, with a significant impact on the quality of life of individuals and their families. Studies have confirmed that individuals with ADHD are at increased risk of comorbid psychiatric disorders (including substance use disorders), accidental injuries, educational underachievement, unemployment, gambling, teenage pregnancy, difficulties socialising, delinquency, suicide and premature death.^3^ Multiple studies have highlighted the substantial economic cost to individuals, families and society.^10,11^ To prevent the negative impact on socio-emotional functioning, financial well-being and quality of life of individuals with ADHD and their families, optimal management – which includes access to diagnosis and treatment – is crucial.

Pharmacotherapy remains the cornerstone of treatment. To date, stimulants remain the best studied and most effective treatment (with an average response rate of 70%) for ADHD.^12^ The majority of international treatment guidelines for ADHD supports the use of stimulants, specifically MPH, as first-line treatment.^4,13,14,15^ Until recently, MPH was the only stimulant available in South Africa. In the public sector, it is still the only, albeit limited, stimulant available. In addition to reducing the core symptoms of ADHD, stimulants improve associated features of ADHD, such as on-task behaviour, academic performance and social functioning, and reduce emotional dysregulation, occupational problems and marital discord.^4^

Medication adherence is a complex issue that affects the successful management of chronic conditions like ADHD. Studies have shown that non-adherence to ADHD pharmacotherapy is generally high – ranging from 13.2% to 64%.^1,16^ There are several reasons why medication adherence can be suboptimal in real-world settings, including socioeconomic factors such as stigma, poverty and low levels of education, patient-related factors such as age and parent and/or family influence, drug-related factors such as side-effects and perceived lack of efficacy and therapy-related factors such as inconvenient dosing regimens and high medication cost.^2,17,18^ Adherence is considered the single most modifiable factor associated with treatment outcomes, yet, evaluations of interventions to improve adherence and persistence in ADHD are lacking.^2^

Non-adherence can be categorised as either unintentional or intentional.^19^ Unintentional non-adherence occurs when the patient is willing to adhere but lacks the necessary resources or ability to do so. Intentional non-adherence occurs when individuals actively choose not to comply with treatment recommendations. The negative impact of unintentional non-adherence can be substantial, with poor symptom control, leading to impaired academic or work performance, social difficulties and a lower quality of life.^20^ Accessibility and affordability of medication pose significant barriers to adherence for individuals with ADHD – the problem being particularly pronounced among those within low socioeconomic environments.^5^

The South African Health Products Regulatory Authority (SAHPRA) classified MPH as a Schedule 6 substance, which indicates a moderate to high potential for abuse or for producing dependence, necessitating close medical management and supervision, and strict control over supply.^6^ This is aligned with the Department of Social Development’s Prevention of and Treatment for Substance Use Disorders Policy which focuses on demand reduction, supply reduction and harm reduction.^21^ Recently, the non-medical use and diversion of stimulants (such as MPH) has received increased attention, with studies reporting prevalence rates of 2.1% – 58.7% and 0.7% – 80.0%, respectively.^22^ In South Africa, diversion among university students has also gained momentum with up to 17% of undergraduate students and 28% of post-graduate medical students using MPH.^23,24^ The majority of these students obtained MPH without a formal diagnostic consultation and script. Therefore, the scheduling status of MPH appears not to be a deterrent in diversion and misuse thereof.

An unintended consequence of the scheduling status of MPH is that it acts as a barrier to treatment for individuals with ADHD. The conditions under which Schedule 6 medicines and substances may be sold or supplied are described in detail in Section 22 A of the 1961 Convention on Narcotic Drugs.^25^ It prohibits the supply of a Schedule 6 substance on a repeat prescription and limits the quantity dispensed to a maximum of 30 days’ supply at the prescribed dose. Accessing monthly scripts from suitably qualified healthcare practitioners is logistically, and financially, challenging.

## Research methods and design

A qualitative analysis of semi-structured interviews with key stakeholders involved in the management of ADHD within South Africa was conducted. The interviews aimed to explore stakeholders’ perceptions and opinions regarding the potential impact of rescheduling MPH on treatment accessibility and adherence.

### Study population and sampling strategy

The target population for this study comprised a diverse group of key stakeholders involved in ADHD management in South Africa. Participants were recruited via the researchers’ professional networks. Purposive sampling was used to invite 50 potential participants representing various stakeholder groups who play a role in addressing the challenges associated with ADHD. Twenty-three stakeholders consented (response rate 46%), while five declined to participate in Zoom-recorded interviews. Although participants identified with more than one stakeholder group (mean 1.6, range 1–3), they were classified according to their primary identification. The sample consisted of nine (39.1%) HCPs, four (17.4%) pharmaceutical industry experts (PIEs), four (17.4%) healthcare funders (HCFs), three (13.0%) individuals with support roles (therapists, parents of children with ADHD, and teachers), two (8.7%) individuals with ADHD and one (4.3%) regulatory expert (RE). Fifteen (60%) of the sample were female.

Sample size is not determined by statistical generalisability but rather by data saturation in qualitative research.^26^ Saturation was reached by the 19th interview when no new themes emerged from the data.

### Data collection

Semi-structured interviews were conducted via Zoom between September 2023 and November 2023. Open-ended questions were asked, with probes where needed to extend answers without leading the desired response, to explore the participants’ perceptions on the benefits and risks associated with the rescheduling of MPH, how to mitigate against potential risks and alternative strategies to enhance accessibility and adherence.

### Data analysis

Recorded interviews were transcribed by the researcher and checked for accuracy by an independent researcher (who was also bound to confidentiality), followed by qualitative analysis using ATLAS.ti (version 23), a computer-aided qualitative data analysis software (CAQDAS) program.^27^ Braun et al.’s approach to reflexive thematic analysis was used by connecting seemingly unrelated and diverse pieces of data into meaningful patterns to generate codes and themes.^28^ The researcher read through the data to obtain an overview and to familiarise herself with the content. Thereafter, dual descriptive-level (level 1 open-coding, where segments of meaning were identified, and level 2 free-coding) and conceptual-level analysis (where related codes were categorised into groups and where relationships between categories were searched for to identify and name themes) followed. The final phase consisted of revisiting the research questions, themes, subthemes and notes to link existing research and literature and assimilate this into the written results and discussion. To protect against researcher bias, the independent researcher randomly checked the primary researcher’s coding and theme formation. A meeting was held to audit the process. The feedback was used to ensure the quality of the set of interviews and analysis thereof. Throughout the research (data collection, analysis and reporting), the researchers practise reflexivity to enhance further trustworthiness of the findings.

### Ethical considerations

Ethical clearance to conduct this study was obtained from the University of Stellenbosch, Social, Behavioural and Education Research Ethics Committee (No. SBS-2023-28787). All participants provided voluntary informed consent for participation and recording of the interviews.

## Results and discussion

**FIGURE 1:**
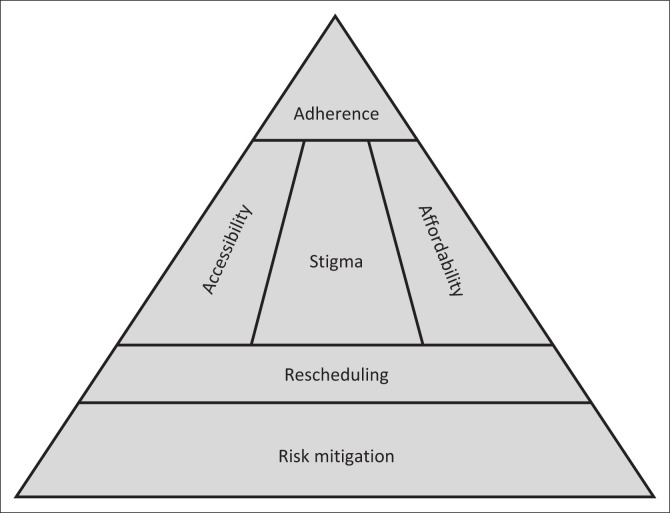
Framework for attention-deficit hyperactivity disorder treatment adherence.

The final code list consisted of 45 codes, representing 351 comments. The codes were then grouped into 11 subthemes, from which six key themes emerged: adherence, accessibility, affordability, stigma, rescheduling of MPH and risk mitigation. Core to these themes is the role of the scheduling of MPH – which can have a protective societal role, but also acts as a barrier to care for individuals with ADHD (see Figure 1).

Results will be reported as (stakeholder group: participant: quote); for example HCP:4:3 refers to healthcare provider, participant 4, quote 3. Abbreviations are reflected as HCP: healthcare provider; PIE: pharmaceutical industry expert; HCF: healthcare funder; PSR: patient support role; PT: patient; RE: regulatory expert.

### Adherence

Participants advocated for extended prescription durations, citing the incongruence between the need for monthly scripts, and the need for optimal treatment of a chronic disorder (PIE:13:7; HCF:15:2). Concerns were raised about inherent symptoms of ADHD which result in administrative challenges to obtaining medication and additional barrier to adherence (HCP:2:3; PT:8:2; HCF:11:1):

‘We know that ADHD comes with executive dysfunction. So putting up all these barriers in order to get the medication that you need to reduce the executive dysfunction kind of feels counterintuitive.’ (PT:8:2)

The importance of treatment adherence to improve outcomes in terms of educational attainment was emphasised – specifically in the public sector where accessing diagnosis and treatment is even more difficult (HCP:2:3; PT:9:5; PSR:12:3; HCP:14:4; HCF:20:4):

‘… they will verbalise to me, “I can’t do this. I haven’t taken my pill.” And when they are on [*them*], these tiny little grade ones will verbalise, “I feel so much better, I can concentrate, it’s so much easier to listen to you and follow instructions.” I mean, it is like life changing for these kids ….’ (PSR:10:11)

Educational success is also considered a safeguard against delinquency (PSR:10:11; PSR:12:14; HCP:14:4; HCP:19:1; HCF:20:4):

‘… if you could get some of the kids on it … what I sort of experienced in my little bit that I have been working there, you will get them out of jail, you’ll keep them out of jail, hopefully [*you*] will keep them from the gangs, because they will do better academically ….’ (HCP:1:11)

There is a bidirectional exacerbating impact of poor adherence and comorbid conditions:

‘We are looking at someone with lowered motivation to do the things they’re needing to do to remain healthy. And even if that medication is providing a sense of relief and productivity and proactivity, we have to look at the entire clinical picture and then we’re looking at someone who is going to neglect their medical needs.’ (HCP:16:56)

Participants expressed their support for the convenience of 6-monthly scripts for obtaining treatment. They indicated that overall adherence and outcomes would improve by reducing administrative challenges such as the need to take time off work and arrange doctors’ visits (PIE:3:3; PIE:5:6; HCP:9:5; HCP:14:4; HCF:22:1).

Our findings are aligned with the previous research which has emphasised the chronic nature of ADHD and the importance of optimal treatment to improve functional outcomes.^3^ The view of participants that adherence is improved by lightening the administrative burden (and enhancing convenience) for individuals with ADHD and their parents, speaks to the modifiable nature of treatment outcomes through simple, practical interventions.^2^

### Accessibility

Participants asserted that the scarcity of specialists constitutes a significant obstacle in accessing a diagnosis and appropriate care (PIE:3:3; HCP:4:9; HCP:14:4; HCF:15:8). Participants reiterated the substantial administrative burden of monthly scripts – not only on individuals with ADHD, but also on HCPs. By improving medical efficiency through reducing the need for monthly scripts, valuable time for patient care could be freed up for a strategic response to a critical resource shortage – specifically, alleviating the pressure in the public sector (HCP:19:1; HCF:15:6; HCP:23:4). However, participants emphasised the need for addressing fundamental issues in access to mental health care services – not only accessibility to medication (HCP:2:13; PSR:10:3; PIE:17:6; RE:18:9).

Participants, acknowledging financial obstacles impeding patient access to care, contemplated whether rescheduling could mitigate these cost-related barriers, especially for those residing in areas a considerable distance from healthcare facilities and providers (HCP:1:6; HCP:6:5; HCP:23:7):

‘I must say in the instance or example where we sit with those patients situated long distances from their healthcare practitioners and to make it much more easier for them … I think that will be the best benefit – to cancel the traveling … still reducing costs for the patient …’ (RE:18:9)

A prevailing theme centred around equity. Broadening access to treatment beyond children and adolescents in private healthcare, to those in the public sector is crucial to address socio-economic disparities. Rescheduling MPH could facilitate access for parents navigating difficulties in monthly clinic visits, particularly in public hospitals or clinics (HCP:4:4; PIE:5:9; HCP:7:8; PSR:10:5; PIE:13:11; HCP:19:10). Improving access to care in the public sector may also be beneficial to the private sector as there will be more pressure on medical schemes to fund treatment (PIE:5:9). However, uncertainty loomed over whether down-scheduling would influence medical schemes’ willingness to cover costs, with prevailing scepticism about overarching financial motivations of pharmaceutical companies (HCP:1:10; PIE:5:10; PT:9:4; HCF:20:1).

Like Petkovic et al.’s study on stakeholder perspectives on medication adherence policies for individuals with chronic conditions, our findings highlight the importance of accessibility of treatment for adherence and improved outcomes.^29^

### Affordability

Participants raised financial aspects as a barrier to access to care and adherence to treatment. Direct costs refer to the amount paid by funders or as out-of-pocket expenses by individuals.^29^ Botha and Schoeman highlighted the lack of medical funding for ADHD in South Africa.^30^ Our participants specifically highlighted the costs associated with frequent script-writing fees and doctors’ visits (HCP:1:1; HCP:2:6; PT:8:12; PT:9:4; HCP:19:2). One HCF specifically mentioned exploitation by practitioners – although it might attest to this participant’s failure to understand out-of-consultation services rendered:

‘Because some of these psychiatrists are also quite ridiculous. You ask them for a new script, and then they want to charge you, you know, some are nice. But others want to charge you every month just to issue a script. Or even if you go to a GP, for a repeat or whatever, they still charge you.’ (HCF:20:4)

Another RE raised the issue of demand for scripts and the (unethical) financial incentives it may provide:

‘They sell these prescriptions. There [*are*] GPs that actually sell this to students because they know there is currently a market. And they make money out of it, because there’s a consultation fee. And if they are dispensing doctors, they are adding on more income on top of that.’ (RE:18:6)

Indirect costs refer to expenses incurred through, for example, care-taking responsibilities, absenteeism from work or additional childcare needed.^31^ The impact of absenteeism from work because of frequent clinic visits, and costs incurred for travelling, has been confirmed in the earlier section on accessibility.

Expenses linked to ADHD were considered prohibitive (PT:8:12; PT:9:3). Our findings concur with an earlier study, which highlighted accessibility as a prerequisite to care and treatment – to prevent the direct and indirect costs of ADHD and the emotional and financial burden that ADHD poses.^32^ Rescheduling MPH, as suggested by participants, may indeed offer financial relief to individuals with ADHD, potentially addressing a critical barrier to access.

### Stigma

The enduring stigma associated with MPH is seen as a persistent barrier to care. Participants proposed that rescheduling might mitigate stigma, counter misinformation about ADHD medications and potentially encourage more individuals to seek and adhere to treatment. The potential impact on parents was emphasised, with rescheduling seen to alleviate fear and reduce perceived complexity in obtaining medication for their children (HCP:2:5; HCP:4:4; PIE:5:9; PT:8:11; PIE:13:9; PSR:21:9). However, participants stressed that parents had entrenched apprehensions about the drug’s safety and efficacy, thus highlighting the challenge of changing deeply ingrained beliefs (HCP:2:1; PT:8:9; PSR:12:4; HCP:16:2; PSR:21:3):

‘[*T*]he general perception is Ritalin is a drug, it’s dangerous, it’s not good, you know, so you still battle with some of your parents to get them to give the medication to their kids.’ (HCP:1:1)

It was also clear that some participants held strong negative opinions regarding MPH – either based on personal narratives or public misconceptions. Several participants raised concerns about the adverse psychological effects of extended MPH use, challenging presumed benefits (PIE:3:1; PT:9:7; HCF:20:2).

‘One thing I didn’t say is that they said that with the prescription of ADHD medication, its actually resulted in more psychological problems with individuals. So, there’s a big study that’s currently going around. It’s that they are prescribed for ADHD but the long-term results are quite bad. People don’t realise that.’ (RE:18:18)

It must be noted that none of these participants were HCPs – which raises the issue of the importance of education. It furthermore serves as a reminder of Thornicroft et al.’s warning: that interventions targeted at only one mechanism at a time would ultimately fail in the absence of fundamental change in addressing both the deeply held attitudes and beliefs about ADHD and the contextual circumstances.^33^

### Rescheduling

Two subthemes arose: the appropriateness of scheduling and the purpose of scheduling.

Firstly, participants questioned the stringent controls on a medication with a long-standing history of safety and efficacy (HCP:7:8; PT:8:11; HCP:14:8). They highlighted the fact that the historical Schedule 6 classification of MPH was rooted in the **injectable** form’s potentially harmful properties (HCP:14:3).

However, some participants were in support of the current scheduling, citing adverse effects such as cardiotoxicity and growth impairment as justification (PIE:13:13; HCP:19:6; PSR:21:4). However, this opinion is not supported by current evidence.^3^

One participant also considered the stringent control imperative to manage the inherent abuse potential of MPH (PIE:17:4). However, this view was not supported by most participants who cited double-blind studies comparing preferences between MPH and other illicit substances among individuals with substance use disorders (HCP:4:11; HCP:16:19). These studies showed that MPH was the least preferred substance with the lowest likelihood of abuse:

‘And you know, everybody’s still on the whole thing, it will be abused. And all of that. I think it’s been very clearly showed that there is not really [*abuse*] … in comparison to other things like cocaine or crack or heroin or any of those others is much less …’ (HCP: 7:4)

Participants emphasised that oral administration, especially of the long-acting formulations, is less likely to cause harm (PIE:3:1; HCP:7:4; HCP:14:3) and less prone to being abused (HCP:1:5; PIE:13:10; HCP:14:6; HCP:19:6).

Secondly, some participants considered the current scheduling as imperative in monitoring individuals with ADHD, especially children (HCP:6:4; PIE:13:13; HCP:15:9; HCP:16:35; RE:18:11). One participant advocated for stringent controls and governance to safeguard this susceptible population, emphasising the need for heightened vigilance and structured governance frameworks:

‘… at the moment, except for the scheduling of methylphenidate, I’m not aware of any additional restrictions, like for instance, I talk about limited access to HCPs, psychiatrists and other specialists … the reality is that there is no restriction on who can prescribe MPH … See how the market has grown for methylphenidate in the last 5 years … as a result, there is a need for perhaps more control and more regulation because the market is more open and more people are taking it.’ (PIE:13:13)

However, good clinical practice, which includes regular monitoring of efficacy and tolerability, is not a function of scheduling – which is primarily focussed at preventing drug abuse and diversion.

Scheduling is also not an effective strategy to prevent diversion and misuse for cognitive enhancement. Some argued that the Schedule 6 classification has not substantially hindered access, acknowledging monthly prescription inconvenience (PIE:13:10; PIE:17:3; HCF:20:9; HCF:22:1), but positing that those genuinely requiring, or wanting, being able to navigate these hurdles despite these control measures (HCP:7:7; PT:9:5; PSR:10:11):

‘I think irrespective what we do about the regulation of the scripts. I mean, they buy it from people, and they get it and they steal it, and they do all sorts of things, and they get it under false pretences. And that’s going to happen whether it’s a Schedule 5 or Schedule 6.’ (HCP:19:7)

This sentiment may not hold for individuals in the public sector who do not have the financial means to access scripts and MPH.

Finally, participants argued that existing prescriber discretion, treating MPH akin to a Schedule 5 medicine and HCPs issuing undated scripts in advance, might make down-scheduling redundant (HCP:1:13; HCP:6:6; PIE:13:7; HCP:14:4; HCF:20:9):

‘So I think that’s one gatekeeping. That could happen, okay, by having that schedule. I think if you down schedule it, of course, then you can get a six month prescription, but the same doctor will need to agree to giving you six month or three month prescription or whatever it might be … the same doctor should be able to exercise the appropriate caution and make an informed clinical decision as to how long I give it to you.’ (PIE:13:7)

### Risk mitigation

Mitigating risks related to potential MPH rescheduling requires a comprehensive and multi-pronged approach. The emphasis on education and supervision underscores the importance of these strategic pillars in addressing systemic disruptions, ensuring ethical conduct, and ultimately contributing to improved patient outcomes.

Participants consider prescribers and pharmacists gatekeepers in the use of MPH, necessitating diligence in both the prescription and dispensing processes (PIE:3:7; PIE:5:3; HCF:11:11; PIE:17:11; HCP:19:11). Participants argued in favour of MPH only to be prescribed by specialist psychiatrists and paediatricians (PIE:3:8; PIE:5:1; HCP:7:2; RE:18:5; PSR:21:7), or, in the case of rescheduling to Schedule 5, only specialists to be able to issue 6-month scripts:

‘But I do think it needs a lot of marketing to go with it to actually explain why it’s being downgraded. And, you know, and to actually make sure that has been really very, very well supervised by somebody who is professional and competent. And I don’t really know if GPs should be prescribing, I do believe that it needs to be a psychiatrist or a pediatrician. I really do. And under those circumstances, I do believe it would be beneficial to everyone.’ (PSR:12:6)

Many participants raised concerns regarding general practitioners not being judicious in making the diagnosis of ADHD (HCP:2:10; PIE:3:1; PIE:5:9; PT:6:8; HCF:11:2; PIE:13:11; HCP:14:9; HCP:16:50; RE:18:13; HCP:19:14; HCF:20:7; PSR:21:7), nor resisting on-demand prescribing (HCP:6:7; PSR:12:4; RE:18:16; HCP:19:3; HCF:20:4). Raising the diagnostic threshold for ADHD, with the initiation of treatment at specialist level, as recommended in the South African guidelines, should be supported.^4^ Participants also highlighted the need for ongoing monitoring and review for both efficacy and tolerability – even if rescheduling enables 6 monthly follow-up consultations (HCP:1:8; PIE:3:6; HCP:6:4; HCP:7:2; HCF:11:2; HCP:16:41).

Recreational use of MPH as a cognitive enhancer is a growing public health concern.^22^ Participants expressed disquiet over increased MPH accessibility leading to increased non-medical use and misuse of MPH in educational and corporate settings (PIE:5:5; PT:9:10; PSR:10:8; PIE:13:5; HCP:16:45; PIE:17:6; RE:18:12; HCP:19:13; HCF:20:3; PSR:21:3). However, to date, current scheduling of MPH has not been a deterrent, with professionals in the medical field seen as major culprits – both in self-prescribing for non-ADHD use, and scripting for others:

‘You know, we actually see that quite a bit in the casualty setting for some odd reason, I think because they work very odd and long hours, you often see the casualty doctor will prescribe Ritalin to four or five of the nursing staff members or working members within the group.’ (PIE:5:5)

Some participants perceived MPH abuse as a societal problem, asserting that it lies beyond the purview of scheduling (HCP:1:15; HCP:2:12; HCP:6:6; PSR:10:8; HCF:20:4). They argue for a public discourse rather than a medical one. Participants also highlighted the challenge of regulating misuse in a culture favouring instant gratification:

‘I don’t think that’s a medical discourse, by the way. I think that’s a public discourse. I think it is this instant gratification, immediate success generation that we have. And unless the population doesn’t say, “We are not tolerating this anymore,” it will not stop.’ (HCP:19:8)

A solution might be to introduce prescription drug monitoring programmes (PDMPs) as a strategy for preventing drug diversion.^34^ Participants called for legislative changes, and an integrated, technologically advanced system featuring robust biometric technology and centralised databases in preventing MPH abuse and diversion (HCP:4:8; PIE:5:8; HCP:6:14; PT:8:13; PIE:17:10). Integrated technology platforms will also enable good governance – with the potential for peer review and drug utilisation reviews (HCF: 15:12).

The key to risk mitigation is education. Participants called for comprehensive campaigns to raise awareness for appropriate diagnosis and management, the societal challenges of misuse and diversion and evidence-based, ethical practice of HCPs – especially general practitioners (HCP:2:13; PIE:3:6; HCP:4:13; HCP:16:54; PIE:17:8; RE:18:4).

## Conclusion

The current Schedule 6 status of MPH is not considered an effective strategy in preventing MPH misuse and diversion but rather acts as a barrier to accessing treatment and treatment adherence. The positive outlook from stakeholders on rescheduling MPH holds significant implications for the ADHD landscape in South Africa.

Stakeholders have identified accessibility, affordability and addressing stigma as drivers of treatment adherence. Rescheduling MPH in the South African context should be explored, with careful consideration of risk-mitigation strategies such as education (especially of gatekeepers such as general practitioners and pharmacists) and technologically supported PDMPs.

A comprehensive, societal-level approach to address the root causes of non-medical use and diversion of MPH is needed as control through the current Schedule 6 status is inefficient. A collaborative initiative with educational and professional institutions to create awareness and implement policies that discourage unreasonable expectations driving the need for non-medical use, is recommended.

A limitation of the study is the potential influence of social desirability bias in stakeholder responses, potentially affecting the authenticity of their perspectives. Future research could employ strategies such as anonymous data-collection methods or triangulation with objective measures to minimise this type of bias. The realised sample did not represent the range of cultural or regional variations in attitudes towards ADHD and its treatment resulting in findings that may not fully encapsulate the potential effects of rescheduling MPH. Future research could explore cultural nuances and regional variations, particularly in a country as diverse as South Africa, to ensure that policy recommendations are culturally sensitive and contextually appropriate.

This study, however, provides contextually relevant insights and suggestions for regulating MPH use. It is crucial to address deeply held attitudes and beliefs to facilitate fundamental change in service delivery and removing structural and practical barriers to treatment adherence.
